# Aligning Predictor-Criterion Bandwidths: Specific Abilities as Predictors of Specific Performance

**DOI:** 10.3390/jintelligence6030040

**Published:** 2018-09-07

**Authors:** Serena Wee

**Affiliations:** 1 School of Psychological Science, University of Western Australia, 35 Stirling Highway, Crawley, WA 6009, Australia; serena.wee@uwa.edu.au; 2 School of Social Sciences, Singapore Management University, 90 Stamford Road, Level 4, Singapore 178903, Singapore

**Keywords:** specific ability, second stratum abilities, academic performance, nested-factor models, relative importance analysis, predictor-criterion bandwidth alignment

## Abstract

The purpose of the current study is to compare the extent to which general and specific abilities predict academic performances that are also varied in breadth (i.e., general performance and specific performance). The general and specific constructs were assumed to vary only in breadth, not order, and two data analytic approaches (i.e., structural equation modeling [SEM] and relative weights analysis) consistent with this theoretical assumption were compared. Conclusions regarding the relative importance of general and specific abilities differed based on data analytic approaches. The SEM approach identified general ability as the strongest and only significant predictor of general academic performance, with neither general nor specific abilities predicting any of the specific subject grade residuals. The relative weights analysis identified verbal reasoning as contributing more than general ability, or other specific abilities, to the explained variance in general academic performance. Verbal reasoning also contributed to most of the explained variance in each of the specific subject grades. These results do not provide support for the utility of predictor-criterion alignment, but they do provide evidence that both general and specific abilities can serve as useful predictors of performance.

## 1. Introduction

Measures of cognitive ability consistently correlate positively with other measures of cognitive ability. Spearman [1] initially argued that these positive correlations among tests (i.e., positive manifold), could be explained by a single, general ability factor, which he termed “*g*”. In contrast, Thurstone [2] emphasized specific abilities in his work, postulating seven specific ability factors. Although the emerging consensus view synthesizes both these extreme positions into a single theoretical framework including both general and specific ability factors [3,4], the debate continues as to the theoretical relations between general and specific abilities [5,6,7,8,9]. The crux of the matter, to paraphrase Humphreys [5] (p. 91), is whether breadth only (as represented by the nested-factors model), or super-ordination also (as represented by the higher-order factor model), defines the general ability factor in relation to the specific ability factors.

As an extension of Spearman’s original unidimensional model of cognitive ability, higher-order factor models assume that the higher-order factor (i.e., general ability) explains the positive correlations among lower-order factors (i.e., specific abilities) [7]. That is, both breadth and superordinate position define the theoretical relations between general and specific abilities. General ability is conceptualized more broadly than specific abilities, and because of its causal status, it is also of a higher order. In contrast, nested-factor models, also referred to as bi-factor models, assume that only breadth distinguishes between general and specific abilities [6,10,11,12]. (When only a few measures of cognitive ability are available, the non-*g* residuals may also be used to reflect specific abilities, in addition to measurement error [13,14].) Although general ability is conceptualized more broadly than specific abilities, it is not assumed to have a causal effect on specific abilities (i.e., they have the same order or position in a hierarchical arrangement) [5,7].

Most of the extant research has been based, implicitly or explicitly, on the assumption that the structure of cognitive abilities is best described by a higher-order factor model. That is, the relationship between a higher-order, general ability factor and a cognitive test variable is fully mediated by the lower-order specific ability factor. To elaborate, empirical tests (e.g., incremental validity analysis using multiple regression) that assign to the general ability factor all the variance that is common among the cognitive ability predictors and the dependent variable, can be argued to be consistent with such a theoretical assumption [8]. For example, in a hierarchical regression analysis, the multiple regression is conducted in steps. At each step, the proportion of variance explained by the predictors (i.e., $R^{2}$) is obtained. Typically, general ability is included in the first step. The $R^{2}$ attributed to general ability in this first step thus includes any of the variance that it shares with the specific abilities (i.e., common variance among cognitive ability predictors is attributed to general ability). Then in the second step, one or more specific abilities are included, and the incremental change in $R^{2}$ between steps is attributed to the specific abilities (i.e., only unique variance is attributed to specific abilities).

Research based on such tests could be interpreted as providing robust evidence for the utility of general ability as a predictor, and at best, only equivocal evidence for the utility of specific abilities as predictors. That is, although general ability (i.e., “*g*”) has been consistently shown to be a useful predictor of practical outcomes such as academic and occupational performance [15,16,17], the utility of specific abilities as predictors of these same outcomes remains hotly contested. Specifically, researchers concluding that there is “not much more than *g*” have highlighted the modest $\left(\Delta R^{2}\approx 0.02\right)$ increments to validity afforded by specific abilities, most especially when a wide range of jobs are being considered [17,18,19]. At the extreme of this position, some have even argued that the continued investigation of specific abilities as predictors is unwarranted, e.g., [20] (p. 341). That said, it should be noted that even small increments in validity can translate into reasonably large practical gains (i.e., dollar utility) [21]. Nonetheless, other researchers have reached seemingly opposite conclusions. For example, when researchers focused on matching specific abilities to the criteria—i.e., perceptual and psychomotor abilities for a job requiring quick and accurate processing of simple stimuli—they found support for the incremental (i.e., unique) validity of specific abilities over *g* [22]. As noted earlier, the nested-factors model provides an alternative conceptualization of the structure of cognitive abilities. In this model, observed test variance is explained by two distinct ability factors: general ability and specific abilities. The general ability factor—distinguished from specific abilities by its breadth—explains variance in a greater number of observed variables than a specific ability factor. However, in this model, general ability is not assumed to cause specific abilities. That is, general ability is not a higher-order factor. Instead, general and specific abilities are all first-order factors. It should be noted that a nested-factors conceptualization of cognitive ability allows for correlations among the general and specific ability factors. However, in practice, when using structural equation modeling (SEM), it is common to assume independence among all the ability factors so as to reduce model complexity and enhance factor interpretability, e.g., [6,7,23]. When independent ability factors are assumed in the nested-factors model, it can be shown to be mathematically equivalent to a higher-order factor model (with additional proportionality constraints) [7].

Research based on a nested-factors model of cognitive ability, has more consistently found support for the utility of specific abilities as predictors, e.g., [6,8,13,14,24,25,26]. In a large sample of middle-school English students, verbal reasoning residuals (obtained by regressing the verbal ability measure on to the general ability measure) significantly predicted standardized exam scores in French [13], and similarly, numerical reasoning residuals significantly predicted national curriculum test scores in math [25]. In a study based on Swedish students, a numeric ability factor was found to correlate strongly with subject grades on a specific science factor [6]. Further, in a meta-analysis based on employed samples, verbal ability was found to account for more of the explained variance in overall job performance (in low-complexity jobs; as compared with a general ability measure) [8]. Similarly, in a sample of military personnel undergoing job-required training in a foreign language, foreign language aptitude was found to account for more of the explained variance (than general ability) in both training course grades, and in a performance-based oral proficiency interview [26].

Thus, previous research based on a nested-factors model of cognitive ability provides support for the utility of specific abilities as predictors of both academic and occupational outcomes. However, these studies differ from each other in at least two important ways: (a) in the alignment of the predictor and criterion bandwidth, and (b) in the data analytic approach used to examine the research question. First, whereas some studies examined the usefulness of specific abilities for predicting specific performance criteria (e.g., [6]), other studies examined their usefulness for predicting general criteria (e.g., [8]). Because some researchers [9,24,27,28,29] have alluded to how a lack of support for specific abilities could have been due to a misalignment between the bandwidth of the predictor and criterion measures, it is important to systematically examine how the alignment of predictor-criterion bandwidths could influence conclusions about the usefulness of cognitive ability predictors. To briefly elaborate on one such example, Wittman and Süß [29] drew on Brunswik’s [30] lens model to develop the concept of Brunswik symmetry, which postulates that “every level of generality at the predictor model has its symmetrical level of generality at the criterion side” [29] (p. 79). And, based on this fundamental assumption, Wittman and Süß [29] therefore predicted that criterion validity is maximized to the extent that the predictor and the criterion are symmetric in their generality (i.e., aligned in the bandwidth of their respective constructs).

Second, these studies also differed in the specific data analytic approach used. Some data analytic approaches have focused on only the unique contribution of predictors as a way of determining the relative importance of general and specific abilities. In contrast, other data analytic approaches, collectively termed as relative importance analyses, have attempted to estimate a predictor’s proportionate contribution to explained variance in the criteria—i.e., to reflect both a predictor’s unique effect and its joint effect when considered with other predictors. As an example of the first type of data analytic approach, studies that have implemented a nested-factors model conceptualization of cognitive abilities using SEM have thus far assumed independence among the ability factors, e.g., [24]. The assumption of independence means that results from these studies will be similar to results obtained when ability is conceptualized using a higher-order factor model, given that these two models are mathematically related (as discussed earlier). That is, the conclusions drawn from both the hierarchical regression analysis (where general ability is entered in the first step of the model, and specific abilities is entered in the second step) or the SEM analysis (where ability factors are constrained to be independent) are likely to indicate the same relative importance ordering of general versus specific ability factors. However, one advantage of the SEM approach over the regression approach, is the ability to control for measurement error.

In the discussion of the SEM approach, the relative importance of a predictor over other predictors in a set is determined by the extent to which that predictor explains unique variance in the criterion. This method for partitioning variance works well if independent predictors are used. If predictors are correlated, as is—at least empirically—the case with cognitive ability predictors, then this approach does not adequately reflect either the direct effect that a specific ability predictor has on the criterion (its correlation with the criterion), nor its joint effect when considered with general ability (because only the unique effect of the predictor on the criterion is considered; common variance among predictors is attributed to general ability). Stated differently, to determine a predictor’s relative importance, one needs to determine its contribution to the common variance in the criterion that has been accounted for by the set of predictors.

This is the problem addressed in the multiple regression literature on relative importance analysis (the second data analytic approach), where several alternative metrics have been developed to supplement the understanding that might be obtained from multiple regression (for a review see [31]): e.g., general dominance weights [32], relative weights [33], and Pratt’s [34] index, with each measure using a slightly different method to measure the relative importance of predictors. *General dominance weights* are obtained by calculating a predictor’s incremental validity for each possible regression submodel in which it could be included, across all the possible submodels. For example, with k=4 predictors, there are $2^{4}-1=15$ possible submodels, and a given predictor is included in eight of these submodels. The general dominance weight reflects a predictor’s relative importance by indexing its overall average incremental validity across submodels, therefore capturing both its contribution to a criterion on its own and jointly with other predictors in the set. *Relative weights* use a different method to partition variance across predictors. Specifically, the *k* correlated predictors are transformed into a new set of *k* variables that are uncorrelated with each other, yet as highly correlated with the original predictors as possible. The criterion can be regressed onto this new set of variables to obtain one set of standardized regression coefficients, and the original variables can be regressed on to this new set of variables to obtain a second set of standardized regression coefficients. Multiplying these two sets of coefficients together therefore provides a measure of the relative contribution of a predictor (on its own and jointly with other predictors) to the variance explained in the criterion. *Pratt’s index*, as an attempt to capture both unique and joint variance explained, is calculated as the product of a predictor’s correlation (i.e., its contribution to explaining criterion variance on its own) and its standardized regression coefficient (i.e., its contribution to explaining criterion variance jointly with other predictors).

In this study, I utilized the relative weights [33] metric. This is because Pratt’s index is not always interpretable (e.g., a negative product moment), and also because it has been shown that rank ordering of predictors in terms of their relative importance tend to be almost identical based on either the general dominance weights or the relative weights. However, relative weights have the added benefit of being computationally easier to obtain [35]. As has been previously highlighted, e.g., [6,8], different data analytic approaches can result in vastly different interpretations, even when using the same data set. It is therefore important to compare data analytic approaches to determine if the same or different conclusions are reached regarding (a) whether specific abilities are useful as predictors, and (b) whether the same specific abilities are identified.

In summary, the purpose of this paper is to examine the utility of specific abilities—in comparison with general ability—for predicting outcomes that are either broadly or narrowly defined. Further, to determine whether differing conclusions on the usefulness of specific abilities as predictors could result from different data analytic approaches, e.g., [8,26], I also compared results obtained from SEM to results obtained from relative weights analysis.

## 2. Materials and Methods

Please see the introductory article for a description of the sample and measures.

### Analytic Strategy

Two data analytic approaches were used to examine the focal research question regarding the relative importance of general versus specific abilities in predicting general versus specific academic performance. In the SEM approach, all models were estimated based on individual-level data and analyzed using Mplus version 7.4 [36] with maximum-likelihood estimation. To evaluate model fit, I considered the incremental fit index provided by the comparative fit index (CFI; [37]), which compares the observed covariance matrix to a baseline model with uncorrelated latent variables, and the absolute fit indices provided by the root mean square error of approximation (RMSEA; [38]) and the standardized root mean square residual (SRMR; [39]). Following the recommendations provided by Hu and Bentler [39], the following cutoff values were used as indicators of good (or acceptable) model fit: CFI > 0.95 (>0.90), RMSEA < 0.06 (<0.08), and SRMR < 0.08.

**Figure 1:**
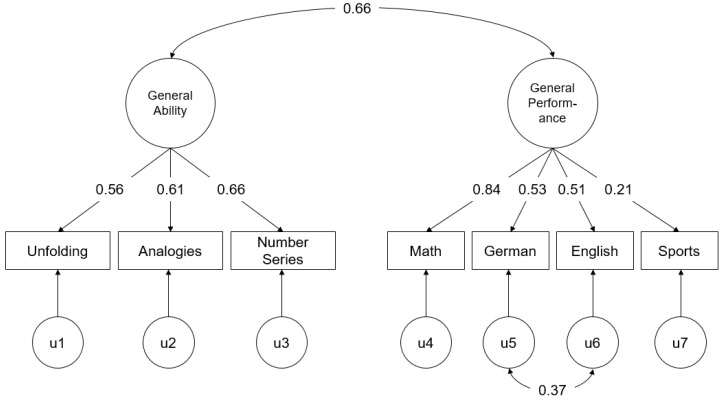
Fully standardized parameter estimates of the structural equation modeling (SEM) model between general ability and general academic performance. All parameter estimates significant at *p* < 0.01.

For these analyses, an initial model was estimated that included only the relationship between a general ability factor and a general academic performance factor. The general ability factor was estimated by all the three cognitive ability tests and the general academic performance factor was estimated by all the four subject grades (χ^2^ = 24.30, *df* = 13, *p* = 0.03, CFI = 0.958, RMSEA = 0.063, SRMR = 0.043). Examination of the modification indices indicated that allowing residuals of the language subjects (i.e., German and English) to be correlated would significantly improve fit, and the initial model was revised accordingly (see Figure 1; χ^2^ = 9.01, *df* = 12, *p* = 0.70, CFI = 1.000, RMSEA = 0.000, SRMR = 0.027). For subsequent models, the residual variance for each indicator (see Figure 1: u1–u7) was used as a measure of the specific ability or specific criterion. Specifically, the unfolding residual variance was used as a measure of spatial reasoning, the analogies residual as a measure of verbal reasoning, the number series residual as a measure of numerical reasoning, and each of the specific subject grade residuals as a measure of specific performance in that subject.

To test the incremental criterion-related validity of general ability for specific academic performance, I examined the validity of general ability for predicting each of the specific subject grade residuals (i.e., u4–u7 in Figure 1), controlling for the relationship between general ability and general academic performance. To test the incremental criterion-related validity of specific abilities for general academic performance, I examined the validity of each specific ability measure (i.e., u1–u3 in Figure 1), controlling for the relationship between general ability and general academic performance. And lastly, to test the incremental criterion-related validity of specific abilities for specific academic performance, I examined the validity of each specific ability measure, for each specific subject grade residual, controlling for the relationship between general ability and general academic performance. In total, 19 separate analyses were conducted, where the focal ability-performance path coefficient was examined. 

In the second data analytic approach, relative weights analyses using ability factor scores were conducted. These factor scores were extracted from the item-level data. For example, the spatial reasoning ability factor score was obtained by fitting a unidimensional factor to the 20 items of the unfolding test, and the general ability factor score was obtained by fitting a unidimensional factor to all the 60 items from the unfolding, analogies, and number series tests. Ability factor scores were used in place of composite scores so as to obtain a general ability measure that was not perfectly collinear with the set of specific ability measures. General academic performance was calculated based on the simple average of all the four subject grades. A separate analysis was conducted for each of the five criteria: general academic performance, and each of the four specific academic performances (as measured by subject grades). The relative weights analyses were conducted based on the individual-level data, in the R statistical software (v. 3.4.1) [40] using the yhat package provided by Nimon, Oswald, and Roberts [41] (see also [42]).

## 3. Results

Table 1 presents the means, standard deviations, and correlations among the cognitive ability and subject grade variables. Correlations among the ability factors were all positive (*M* = 0.56; range: 0.32 to 0.86), with the highest correlations being between general ability and the specific abilities. Correlations among the specific subject grades were also all positive (*M* = 0.46; range: 0.11 to 0.79), with the highest correlations being between general academic performance and the specific subject grades. As expected, cognitive ability scores were positively related to subject grades (*M* = 0.23; range: −0.01 to 0.42), with the exception of the relationship between spatial reasoning (i.e., unfolding) and sports grades (*r* = −0.01, *p* > 0.05).

Results from the SEM analysis are presented in Table 2. Controlling for the relationship between general ability and general academic performance (*r* = 0.66, *p* < 0.01; see Figure 1), the standardized path coefficients between the specific ability residuals and (general or specific) academic performance were estimated. After controlling for the relationship between general ability and general academic performance, none of the specific ability residuals significantly predicted general academic performance: unfolding = −0.06, analogies = 0.12, and number series = −0.09 (all *p*s > 0.05). General ability also did not significantly predict specific academic performance (i.e., subject grade residuals): math = 0.19, German = 0.00, English = 0.02, and sports = −0.016 (all *p*s > 0.05). And lastly, none of the specific ability-specific academic performance relationships were significant, after controlling for the relationship between general ability and general performance (*M* = 0.00; range: −0.10 to 0.08). Thus, these results do not provide support for the utility of specific ability predictors, after the taking into account the relationship between general ability and general academic performance.

Table 3 presents the results for the relative weights analysis (i.e., raw and scaled weights) for general academic performance and for each specific academic grade. For ease of comparison with traditional regression-based metrics, it also presents the correlation, and standardized and unstandardized regression coefficients. Besides the correlation coefficient, all other metrics were obtained from regression models that included all four ability predictors.^1^ Overall, the variance accounted for by ability predictors was 18.4% in general academic performance, 21.9% in math grades, 8.4% in German grades, 10.0% in English grades, and 1.1% in sports grades. Of the four ability predictors, general ability showed the strongest correlation with general academic performance (*r* = 0.37). Based on the bootstrapped 95% CI of the difference between pairs of values, this correlation is significantly stronger than the correlations between general academic performance with either unfolding (*r* = 0.25), or number series (*r* = 0.30), but not with analogies (*r* = 0.35). When ability predictors are considered jointly (i.e., regression coefficients) unfolding (*b* = 0.81), analogies (*b* = 0.73), and number series (*b* = 1.16) provide unique, positive contributions to variance explained in general academic performance. Also, general ability is now negatively related to general academic performance (*b* = −1.74), making these regression results somewhat difficult to interpret. In contrast, the relative weights capture both a predictor’s unique and shared contribution to explaining variance in the criterion. General ability contributed to 3.7% of the explained variance in general academic performance, and hence contributed to 20.1% (=0.037/0.184) of the total explained variance in general academic performance. Similarly, unfolding, analogies, and number series contributed to 15.7%, 40.8%, and 23.4% of the total explained variance in general academic performance, respectively. Thus, relative weights indicated that, when a predictor’s shared and unique contribution to the explained variance in the criterion were considered simultaneously, verbal reasoning (i.e., analogies) was found to be a more important predictor of general academic performance than was general ability (i.e., 40.8% vs. 20.1%). However, the bootstrapped 95% CI of the difference between the raw weights indicate that this difference is not statistically significant.

Based on the relative weights, verbal reasoning (i.e., analogies) was also the most important predictor for math (30.7%), German (38.1%), English (51.0%), and sports (54.5%) grades. That is, it contributed to a greater proportion of total explained variance than did general ability in each subject grade: math (21.6%), German (20.2%), English (19.0%), and sports (18.2%). Although these results may be consistent with expectations for German and English grades—i.e., in addition to contributing to the shared variance explained, verbal reasoning also contributed uniquely to performance in language-based subjects—the obtained results are somewhat surprising for math and sports grades. For both these subjects, although verbal reasoning was found to be the most important predictor, numerical reasoning (i.e., number series) was ranked second in importance: math (26.6%) and sports (27.3%). However, as with the results for general academic performance, bootstrapped 95% CIs indicated that none of the differences between raw weights are statistically significant. Lastly, general ability accounted for about 20% of the explained variance in the various performance criteria, which means that, taken together, the specific abilities accounted for about 80% of total explained variance in the performance criteria. These results suggest that specific abilities (especially verbal reasoning) are useful predictors of both general and specific academic performance.

## 4. Discussion

In order to advance the discussion on the usefulness of general and specific abilities for predicting performance, this study examined the validity of these abilities when predicting broadly versus narrowly defined criteria. The SEM approach identified general ability as the strongest (and only) predictor of general academic performance; it explained 44% of the variance in general academic performance. In contrast, the relative weights analysis identified verbal reasoning (i.e., analogies) as a more important predictor of general academic performance than even general ability. Specifically, of the 18% of the variance jointly accounted for by the ability predictors, general ability’s proportionate contribution was 20% while verbal reasoning’s proportionate contribution was double this at 41%. These results are consistent with much of the previous literature. As reviewed in the introduction, the SEM approach consistently identifies general ability as an important predictor of broadly defined criteria, e.g., [24], whereas several studies based on the relative weights approach have identified verbal ability/verbal reasoning as the most important predictor of broadly defined criteria such as overall job performance (at least in low complexity jobs) [8] and training grades [26].

Further, the SEM approach indicated that neither general nor specific abilities significantly predicted specific academic performance (i.e., subject grade residuals), once the relationship between general ability and general academic performance was accounted for. In contrast, relative weights analysis identified verbal reasoning as the most important predictor for each of the specific subject grades. In sum, at least based on these data, these results do not provide evidence for the utility of aligning predictor and criterion bandwidth for maximizing validity. Instead, these results suggest that, although general and specific abilities can serve as useful predictors of performance, conclusions regarding their utility depended critically on the data analytic approach used. 

There are several plausible explanations for the differences in the pattern of results across approaches. Although both data analytic approaches were based on a nested-factors conceptualization of the cognitive abilities, the ability constructs were still operationalized differently across approaches. In the SEM approach, the general and specific abilities were constrained to not share any variance, whereas in the relative weights analysis, cognitive abilities were allowed to correlate with one another. Thus, to the extent that general and specific abilities are actually correlated, the SEM model is therefore mis-specified and the accuracy of our conclusions regarding the utility of general versus specific abilities reduced. For example, one possible way that general and specific abilities could be correlated is if multiple, discrete cognitive processes interact dynamically, resulting in an emergent, observed positive manifold across cognitive tests (i.e., general ability). Based on this theoretical mechanism, the correlation between general ability and a specific ability (e.g., verbal reasoning) occurs to the degree that the specific ability results from the interactions over time of a subset of the cognitive processes that are also involved in the emergence of the general factor. The relative weights analysis does not require independent predictors, and therefore is able to more accurately capture the proportionate contribution of individual predictors to explaining variance in the criteria. However, even though relative weights analysis was developed specifically to determine the relative importance of correlated predictors, the method is still based on multiple regression. Therefore, it does not remove the underlying issue of multicollinearity (when it exists). In this dataset, for example, general ability was quite highly correlated (*r*s > 0.70) with the specific abilities. As a consequence, confidence intervals around the point estimates are also fairly wide. Thus, although verbal reasoning was identified as contributing more than general ability to explained variance across all criteria, the difference in these raw relative weights (for each criterion) was not statistically significant at *p* < 0.05.

Perhaps just as importantly, the data analytic approaches also differed in how the performance constructs were operationalized. Whereas the SEM analyses used specific performance measures that excluded general performance variance, the relative weights analyses used specific performance measures that included both general and specific performance variance. Further, it should be noted that a unidimensional model of performance (with correlated language grade residuals) fit the data extremely well ($\chi_{\left(12\right)}^{2}$ = 9.01, *p* = 0.70). This suggests that academic performance is adequately described by just a single performance factor; the specific subject grade residuals might not have served as adequate or reliable indicators of specific subject grade performance, once variance associated with general academic performance was removed.

Taken together, these results show that data analytic approaches can have implications as to which specific abilities are identified as useful predictors of specific performance criteria. Thus, this research suggests that even when data analytic approaches are based on the same theoretical assumptions (in this case, based on the nested-factor model of cognitive abilities) it is still possible that substantively different conclusions regarding specific abilities can be reached. Consequently, future research efforts should be directed toward better understanding how data analytic approaches can impact our conclusions regarding the usefulness of a given specific ability predictor. 

### Limitations and Future Research Directions

A number of study limitations should be noted. First, and perhaps most critically, only a small number of measures were available for the cognitive ability predictors, and for the performance criteria. Even if it could be reasonably argued that the general ability and general performance constructs were adequately captured by these measures, this argument is unlikely to extend to the construct-valid assessment of either the specific ability or specific performance constructs. That is, in this study, across both data analytic approaches, measures of the specific constructs included both specific construct variance, as well as error variance. Stated differently, unreliable measures diminish our ability to derive useful and interpretable specific factors [43,44]. In turn, because general and specific ability predictors differ in how reliably they are measured, this obfuscates our ability to meaningfully evaluate their usefulness as predictors.

Second, it should be noted that a substantial portion of the variance in general and specific academic performance was unexplained by cognitive ability. This is most notable, for example, with the specific performance criterion of sports grades, where general and specific abilities together explained only 1.1% of the variance in the criterion. This suggests that non-cognitive individual difference constructs (such as interests, personality, or motivation) or group difference variables (such as sex or race) also have a role to play in predicting academic performance. Specifically, theoretical arguments regarding the interplay of interests and motivations in determining domain-relevant specific abilities (i.e., knowledge and skills), e.g., [45,46], as well as empirical research demonstrating how interests and abilities are mutually causal over time [47], suggest that a fruitful avenue for better understanding the usefulness of specific abilities for predicting consequential outcomes resides in disentangling the dynamic relationships between specific abilities and specific interests, as they jointly predict performance over time.

This paper examined the utility of aligning the bandwidth of predictors to criteria. Although no support was found for the utility of alignment, this might have been because the previously identified limitations did not allow this proposition to be adequately tested. Further, this study also highlights the value of explicitly considering the criterion when evaluating the usefulness of cognitive ability predictors. Because there are important practical criteria (beyond performance) that relate to cognitive abilities, an evaluation of the predictive utility of cognitive abilities should also consider these other criteria (e.g., sex, race and adverse impact potential) in addition to, or in conjunction with, performance. For example, research by Wee, Newman, and Joseph [48] demonstrated that the use of specific abilities, rather than general ability, could improve an organization’s diversity outcomes, even whilst maintaining expected job performance at levels that would be obtained from a general ability predictor. 

Lastly, in this paper, positive manifold was taken as evidence that there is a general ability factor, i.e., a common cause that provides a parsimonious account for a substantial portion of the variance in cognitive ability measures. However, there are several plausible explanations for how observed variables could be positively correlated even in the absence of such an underlying, causal general factor [49,50,51,52,53]. Although a general ability construct provides an extremely effective and efficient predictor of performance across a wide variety of domains [15,16,17], it does not appear to have significantly advanced our understanding of the manner in which cognitive ability relates to important practical outcomes (i.e., “*g* is poorly defined and poorly understood” [54], p. 3). A set of less parsimonious—but more substantively interpretable—specific abilities could provide the alternative required to develop a better articulated theory of how cognitive ability relates to practical outcomes, and in so doing, further enhance the value of specific abilities as predictors of these same outcomes.

## Figures and Tables

**Table 1:** Descriptive statistics for the overall sample (*N* = 219).

Variable	1.	2.	3.	4.	5.	6.	7.	8.	9.
1. General Performance	--								
2. Math	0.787	--							
3. German	0.775	0.439	--						
4. English	0.757	0.427	0.542	--					
5. Sports	0.442	0.183	0.156	0.108	--				
6. General Ability	0.372	0.424	0.261	0.251	0.029	--			
7. Unfolding	0.252	0.310	0.213	0.132	−0.014	0.732	--		
8. Analogies	0.348	0.348	0.236	0.272	0.066	0.652	0.321	--	
9. Number Series	0.300	0.349	0.185	0.212	0.033	0.865	0.397	0.392	--
*M*	4.127	3.808	3.913	3.735	5.050	0.000	0.000	0.000	0.000
*SD*	0.666	1.153	0.937	0.940	0.718	0.950	0.913	0.887	0.933

Note: Correlations ≥|0.14| are statistically significant at *p* < 0.05.

**Table 2:** Standardized relationship between cognitive abilities and academic performance.

Variable	General Performance	Math (res)	German (res)	English (res)	Sports (res)
General Ability	--	0.19	0.00	0.02	−0.16
		(0.24)	(0.13)	(0.12)	(0.15)
Unfolding	−0.06	0.02	0.07	−0.09	−0.10
	(0.12)	(0.08)	(0.06)	(0.07)	(0.07)
Analogies	0.12	−0.02	−0.01	0.08	0.01
	(0.12)	(0.10)	(0.07)	(0.06)	(0.08)
Number Series	−0.09	0.04	−0.05	0.02	−0.09
	(0.19)	(0.10)	(0.07)	(0.07)	(0.08)

Note: Analyses were conducted separately for each of the 19 standardized path coefficients, controlling for the relationship between general ability and general academic performance. res = residual, i.e., the residual variance after removing variance due to general ability or general academic performance. Standard errors are reported in parentheses.

**Table 3:** Regression-based metrics of predictor variable importance.

Metric	General Ability	Unfolding	Analogies	Number Series
**General Performance** $\left(R^{2}=\;0.184\right)$				
*r*	0.372 (0.232, 0.497) ^a, b^	0.252 (0.121, 0.382) ^a^	0.348 (0.223, 0.472)	0.300 (0.155, 0.431) ^b^
*b*	−1.745 (−3.303, 0.014) ^a, b, c^	0.814 (0.113, 1.450) ^a^	0.732 (0.171, 1.258) ^b^	1.163 (0.090, 2.083) ^c^
*B*	−2.489 (−4.658, 0.018) ^a, b, c^	1.115 (0.166, 1.964) ^a^	0.976 (0.235, 1.684) ^b^	1.628 (0.131, 2.869) ^c^
Raw weight	0.037 (0.020, 0.068)	0.029 (0.006, 0.070)	0.075 (0.025, 0.151)	0.043 (0.012, 0.094)
Scaled weight	20.109%	15.761%	40.761%	23.370%
**Math Performance** $\left(R^{2}=\;0.219\right)$				
*r*	0.424 (0.302, 0.545) ^a, b^	0.310 (0.175, 0.436) ^a^	0.348 (0.222, 0.472)	0.349 (0.216, 0.480) ^b^
*b*	−3.133 (−5.604, −0.360) ^a, b, c^	1.521 (0.380, 2.512) ^a, d^	1.262 (0.344, 2.063) ^a, e^	2.130 (0.453, 3.656) ^c, d, e^
*B*	−2.580 (−4.690, −0.295) ^a, b, c^	1.204 (0.311, 2.036) ^a, d^	0.971 (0.269, 1.587) ^a, e^	1.722 (0.360, 2.942) ^c, d, e^
Raw weight	0.047 (0.028, 0.082)	0.046 (0.014, 0.099)	0.067 (0.022, 0.141)	0.058 (0.021, 0.119)
Scaled weight	21.560%	21.101%	30.734%	26.606%
**German Performance** $\left(R^{2}=\;0.084\right)$				
*r*	0.261 (0.132, 0.382) ^a^	0.213 (0.077, 0.350)	0.236 (0.101, 0.350)	0.185 (0.052, 0.319) ^a^
*b*	−1.021 (−3.478, 1.376)	0.566 (−0.478, 1.621)	0.496 (−0.277, 1.287)	0.681 (−0.780, 2.094)
*B*	−1.035 (−3.396, 1.478)	0.551 (−0.487, 1.566)	0.469 (−0.253, 1.222)	0.678 (−0.760, 2.074)
Raw weight	0.017 (0.008, 0.042)	0.022 (0.003, 0.074)	0.032 (0.005, 0.081)	0.013 (0.002, 0.047)
Scaled weight	20.238%	26.190%	38.095%	15.476%
**English Performance** $\left(R^{2}=\;0.100\right)$				
*r*	0.251 (0.114, 0.387) ^a^	0.132 (0.001, 0.273) ^a^	0.272 (0.135, 0.391)	0.212 (0.074, 0.360)
*b*	−1.907 (−4.060, 0.475)	0.817 (−0.160, 1.755)	0.828 (0.081, 1.565)	1.268 (−0.093, 2.554)
*B*	−1.927 (−4.169, 0.470)	0.793 (−0.153, 1.708)	0.781 (0.069, 1.464)	1.258 (−0.089, 2.546)
Raw weight	0.019 (0.008, 0.045)	0.006 (0.001, 0.038)	0.051 (0.010, 0.115)	0.024 (0.004, 0.069)
Scaled weight	19.000%	6.000%	51.000%	24.000%
**Sports Performance** $\left(R^{2}=\;0.011\right)$				
*r*	0.029 (−0.102, 0.159)	−0.014 (−0.145, 0.116)	0.066 (−0.072, 0.198)	0.033 (−0.105, 0.164)
*b*	−0.921 (−2.675, 0.882)	0.352 (−0.401, 1.110)	0.345 (−0.206, 0.912)	0.571 (−0.496, 1.648)
*B*	−1.217 (−3.620, 1.131)	0.447 (−0.521, 1.476)	0.426 (−0.259, 1.106)	0.742 (−0.657, 2.180)
Raw weight	0.002 (0.001, 0.020)	0.000 (0.000, 0.020)	0.006 (0.000, 0.041)	0.003 (0.000, 0.029)
Scaled weight	18.182%	0.000%	54.545%	27.273%

Note: *r* = correlation coefficient, *b* = standardized regression coefficient, *B* = unstandardized regression coefficient, Raw weight = raw relative weight, Scaled weight = rescaled relative weight (sums to 100 within row). Bootstrapped 95% CI are presented in parentheses. Subscripts (i.e., a–e) indicate statistically significant differences in pairs of metrics (within rows).

## Footnotes

1 These results are supplemented by a hierarchical regression analysis showing the incremental contribution (over general ability) of each specific ability by itself, in a pair, and in a triplet (see Appendix A Table A1).

## Appendix A

**Table A1:** Hierarchical regression analysis.

Subset	*R* ^2^	Unfolding (U)	Analogies (A)	Number Series (N)
**General Academic Performance**				
General Ability (G)	0.138	0.001	0.019	0.002
G,U	0.139		0.019	0.008
G,A	0.157	0.000		0.001
G,N	0.140	0.007	0.018	
G,U,A	0.158			0.026
G,U,N	0.147		0.037	
G,A,N	0.158	0.026		
G,U,A,N	0.184			
**Math Performance**				
G	0.180	0.000	0.009	0.001
G,U	0.180		0.010	0.002
G,A	0.189	0.001		0.000
G,N	0.181	0.001	0.008	
G,U,A	0.190			0.029
G,U,N	0.182		0.037	
G,A,N	0.189	0.030		
G,U,A,N	0.219			
**German Performance**				
G	0.068	0.001	0.008	0.006
G,U	0.069		0.010	0.006
G,A	0.076	0.004		0.002
G,N	0.074	0.001	0.003	
G,U,A	0.079			0.005
G,U,N	0.075		0.009	
G,A,N	0.078	0.006		
G,U,A,N	0.084			
**English Performance**				
G	0.063	0.006	0.020	0.000
G,U	0.069		0.016	0.007
G,A	0.083	0.001		0.004
G,N	0.063	0.013	0.024	
G,U,A	0.084			0.016
G,U,N	0.076		0.024	
G,A,N	0.087	0.013		
G,U,A,N	0.100			
**Sports Performance**				
G	0.001	0.003	0.004	0.000
G,U	0.003		0.002	0.001
G,A	0.005	0.001		0.002
G,N	0.001	0.003	0.006	
G,U,A	0.006			0.005
G,U,N	0.004		0.007	
G,A,N	0.007	0.004		
G,U,A,N	0.011			
